# Influence of inflammatory and reninangiotensin system gene polymorphisms ACE2 rs2285666, IL1A rs1800587, and TNF rs1800629 on COVID-19 severity and the persistence of symptoms in the post-COVID-19 phase: a cross-sectional study

**DOI:** 10.31744/einstein_journal/2026AO1503

**Published:** 2026-05-21

**Authors:** Matheus Daudt-Lemos, Evelyn Maciel de Oliveira, Alice Ramos-Silva, Natalia Fonseca Rosário, Thays Araújo Gonçalves, Camila de Melo Carvalho Nascimento, Amanda Mendes do Valle, Lialyz Soares Pereira André, Fabio Aguiar-Alves, Jorge Paulo Strogoff de Matos, Jocemir Ronaldo Lugon, Jorge Reis Almeida, Thalia Medeiros, Fabiana Barzotto Kohlrausch, Andrea Alice Silva

**Affiliations:** 1 Universidade Federal Fluminense Multiuser Laboratory for Research Support in Nephrology and Medical Sciences School of Medicine Niterói RJ Brazil Multiuser Laboratory for Research Support in Nephrology and Medical Sciences (LAMAP), School of Medicine, Universidade Federal Fluminense, Niterói, RJ, Brazil.; 2 Universidade Federal Fluminense Graduate Program in Pathology Faculdade de Medicina Niterói RJ Brazil Graduate Program in Pathology, Faculdade de Medicina, Universidade Federal Fluminense, Niterói, RJ, Brazil.; 3 Universidade Federal Fluminense Postgraduate Program in Medical Science Faculdade de Medicina Niterói RJ Brazil Postgraduate Program in Medical Science, Faculdade de Medicina, Universidade Federal Fluminense, Niterói, RJ, Brazil.; 4 Universidade Federal Fluminense School of Pharmacy Molecular Epidemiology and Biotechnology Laboratory Niteroi RJ Brazil Molecular Epidemiology and Biotechnology Laboratory, School of Pharmacy, Universidade Federal Fluminense, Niteroi, RJ, Brazil.; 5 Palm Beach Atlantic University Lloyd L. Gregory School of Pharmacy Department of Pharmaceutical Sciences FL United States Department of Pharmaceutical Sciences, Lloyd L. Gregory School of Pharmacy, Palm Beach Atlantic University, FL, United States.; 6 Universidade Federal Fluminense Institute of Biology Department of General Biology Niterói RJ Brazil Human Genetic Laboratory, Department of General Biology, Institute of Biology, Universidade Federal Fluminense, Niterói, RJ, Brazil.

**Keywords:** Uromodulin, Angiotensin-converting enzyme 2, Cytokines, Post-acute COVID-19 syndrome, olymorphism, genetic, SARS-CoV-2, COVID-19

## Abstract

**Objective:**

We evaluated the influence of single-nucleotide polymorphisms in cytokine, renin-angiotensin-aldosterone system, and uromodulin genes on COVID-19 severity and the persistence of symptoms in the post-COVID phase.

**Methods:**

Two cross-sectional cohort studies were conducted: a retrospective cohort (cohort 1) from early phase of the pandemic and a prospective cohort (cohort 2) including patients with symptoms in the post-COVID phase. Single-nucleotide polymorphism detection was performed using real-time and conventional polymerase chain reaction.

**Results:**

Cohort 1 included 112 patients (mean age 57.4±17.5 years, 42% male). *ACE* rs4646994, *ACE2* rs2285666, *IL1A* rs1800587, and *TNF* rs1800629 were associated with COVID-19 severity. However, when evaluating more specific outcomes such as intensive care unit admission and the need for invasive mechanical ventilation, associations were observed only for *ACE2* and *TNF*. In women, the *ACE2* rs2285666 GG genotype (p=0.003) and G allele (p=0.013) were associated with intensive care unit admission. In addition the A allele of *TNF* rs1800629 was associated with a higher risk of invasive mechanical ventilation (p<0.001). Cohort 2 included 107 patients (mean age 54.7±15.18 years 27.2% male). The *TNF* GA genotype was a risk factor for cough (p=0.03) and exertional fatigue (p=0.049). Lastly, *IL1A* rs1800587 was associated with the risk of persistent respiratory symptoms.

**Conclusion:**

Our results suggest that single-nucleotide polymorphisms in *ACE2, IL1A,* and *TNF* may be associated with an increased risk of severe COVID-19 and persistence of symptoms in affected patients.

## In Brief

This study investigated whether polymorphisms in ACE2, IL1A, and TNF genes are associated with COVID-19 severity and post-COVID symptoms in Brazilian patients. Our findings showed specific variants were associated with intensive care unit admission, mechanical ventilation, and persistent respiratory symptoms.

## Highlights

■ ACE2 rs2285666 GG genotype increased the risk of intensive care unit risk in women.
■ TNF rs1800629 A allele was associated with severe COVID-19 and invasive mechanical ventilation.
■ IL1A rs1800587 influenced disease severity and respiratory symptoms.
■ TNF polymorphism was associated with post-COVID, cough and fatigue.

## Graphical abstract

**Figure:**
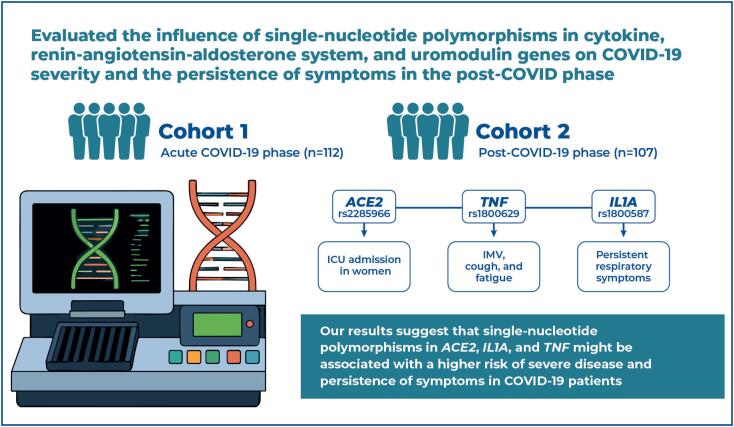

## INTRODUCTION

Severe acute respiratory syndrome coronavirus 2 (SARS-CoV-2) infection is known to impact the immune response,^(1)^ and single-nucleotide polymorphisms (SNPs) in key genes related to host immunity and cell function contribute to the clinical variability observed in coronavirus disease 2019 (COVID-19).^(2-4)^ Notably, exacerbation of the immune response to SARS-CoV-2 involves the secretion of various immune mediators, which are crucial in determining the severity of COVID-19.^(2,3,5)^ While most individuals with COVID-19 recover without complications, a subset may prolonged symptoms or sequelae, often referred to as post-COVID-19 condition (PCC).^(6)^ According to the World Health Organization (WHO), PCC is a clinical condition characterized by the persistence of symptoms or the development of new symptoms three months after SARS-CoV-2 infection, lasting at least two months and not explained by an alternative diagnosis.^(7)^ The most common persistent symptoms of PCC have been identified, and several mechanisms to explain its pathophysiology, such as an association with a specific inflammatory profile, are currently under investigation.^(8)^

Refining the existing diagnostic approaches and exploring alternative factors contributing to the persistence of COVID-19 symptoms beyond the acute infection phase remain crucial areas of research. Host SNPs are thought to play a significant role in determining an individual's susceptibility or resistance to various viral infections, and numerous studies have investigated genetic polymorphisms in the context of COVID-19 to date.^(4,9-11)^ Certain studies have indicated that SNPs in genes involved in innate and adaptive immune responses, as well as in viral binding and host cell entry, are associated with the development and severity of COVID-19.^(12,13)^ Nevertheless, the specific SNPs that contribute to susceptibility, prognosis, and the persistence of symptoms in the post-acute COVID-19 phase remain to be fully elucidated,^(14-16)^ particularly given the variability in genetic profiles across different populations.^(9,10)^ The SNPs selected in this study were chosen based on their functional roles in the main immunological and physiological pathways relevant to COVID-19 pathogenesis. Variants in cytokine genes — such as *TNF* (rs1800629), *IL10* (rs1800871, rs1800896), *IL6* (rs1800795), and *IL1A* (rs1800587) — influence the expression of pro- and anti-inflammatory mediators involved in the acute response to viral infections and chronic inflammation.^(17-21)^ Polymorphisms in the angiotensin-converting enzyme genes - *ACE* (rs4646994) and *ACE2* (rs2285666) - affect the expression of proteins essential for viral entry and regulation of the renin-angiotensin system, which has been implicated in disease severity.^(22-24)^ In addition, SNPs in the *UMOD* gene (rs12917707, rs13333226, rs4293393) modulate uromodulin levels, increase *UMOD* gene expression, and are associated with kidney function, a frequent site of COVID-19-related complications.^(25-27)^ Therefore, we hypothesize that these SNPs may influence the medium- and long-term pathophysiology of patients with COVID-19.

## OBJECTIVE

The aim of this study was to evaluate the influence of SNPs in different genes, including *IL1A* (rs1800587), *IL6* (rs1800795), *IL10* (rs1800896 and rs1800871), *TNF* (rs1800629), *ACE* (rs4646994), *ACE2* (rs2285666) and *UMOD* (rs4293393, rs13333226 and rs12917707), on COVID-19 severity in a group of Brazilian patients during the first wave of the pandemic and in the post-acute phase of the disease, as well as to investigate associations with the development of the post-COVID-19 condition and its clinical manifestations.

## METHODS

### Ethical approval

The present study was conducted in two phases, each approved by a Research Ethics Committee The acute phase of COVID-19 was approved by the Research Ethics Committee of Universidade Federal Fluminense (CAAE: 30623520.5.0000.5243; # 3.987.966) and the post-COVID phase was approved by the Research Ethics Committee of *Fundação Carlos Chagas Filho de Amparo à Pesquisa do Estado do Rio de Janeiro* (CAAE: 59213722.5.0000.5243; # 5.494.515). In accordance with ethical standards, written informed consent was obtained from all participants.

### Patients and study design

For cohort 1, we conducted a retrospective cross-sectional study including 112 adult patients enrolled from April to October 2020, diagnosed with acute SARS-CoV-2 infection by RT-PCR. All patients were hospitalized at the *Hospital Universitário Antônio Pedro* of the *Universidade Federal Fluminense* (HUAP/UFF/EBSERH). For cohort 2, we conducted a prospective study involving 107 adult patients in the post-COVID-19 period, followed up at the same hospital since July 2022, to diagnose PCC. All participants in this cohort had at least one positive RT-PCR test result for SARS-CoV-2. Patients with missing laboratory or clinical data that could hinder data analysis, as well as those with kidney failure requiring dialysis, were excluded.

Clinical and laboratory data from the acute infection period were obtained from medical records. Patients presenting with fever, cough, sore throat, headache, myalgia, nausea, vomiting, diarrhea, loss of taste and smell, oxygen saturation ≥94%, and no abnormalities on imaging tests were classified as having mild/moderate disease. Those with oxygen saturation <94% on ambient air, pulmonary infiltrates involving >50% of the lungs, septic shock, thrombotic disease, and/or multiple organ dysfunction, or requiring invasive mechanical ventilation (IMV) and intensive care unit (ICU) admission due to respiratory failure, were classified as having severe disease.^(28,29)^

Patients presenting with dyspnea but maintaining oxygen saturation above 94% were classified as having moderate disease, whereas those without dyspnea and with oxygen saturation above 94% were classified as having mild disease.^(30)^

Post-COVID-19 condition was diagnosed according to WHO criteria (persistence or development of new symptoms three months after the initial SARS-CoV-2 infection, lasting at least two months and not explained by an alternative diagnosis).^(7)^

### Molecular tests for single-nucleotide polymorphism characterization

High-molecular-weight genomic DNA was isolated from venous blood using the QIAamp^®^ DNA Mini Kit (QIAGEN, Germany). Genotyping of *IL1A* (rs1800587), *IL6* (rs1800795), *IL10* (rs1800896 and rs1800871), *TNF* (rs1800629), *ACE2* (rs2285666) and *UMOD* (rs4293393, rs13333226 and rs12917707) was performed using real-time Polymerase Chain Reaction (PCR) with predesigned and validated Taqman^®^ assays (C___9546481_30, C___1839697_20, C___1747360_10, C___1747362_10, C___7514879_10, C___2551626_1_, C__27865986_10, C__31122293_10 and C__31122302_20, respectively; Thermo Fisher Scientific, Brazil), according to the manufacturer's instructions. Importantly, patients with chronic kidney disease (CKD) diagnosed before admission were excluded from the *UMOD* (rs4293393, rs13333226, and rs12917707) analysis. Furthermore, the genotyping of *ACE* (rs4646994) was performed by standard PCR. For this gene, we analyzed an INDEL polymorphism (I/D) insertion/deletion (I/D) polymorphism, in wich the alleles consisted of a 190-bp fragment (D allele) and a 490-bp fragment (I allele), detected by electrophoresis of PCR products on a 2% agarose gel. PCR conditions and primers were previously described by Mohebbi et al.^(31)^

### Statistical analysis

Allele frequencies were obtained by gene counting. Deviations from the Hardy-Weinberg equilibrium were evaluated using the *χ*^2^ test. The haplotype block structure of the SNPs in *IL10* and *UMOD* was examined using the spine of linkage disequilibrium (LD) as implemented in Haploview.^(32)^ Categorical variables were expressed as absolute and relative frequencies and compared using the *χ*^2^ or Fisher's exact tests. Continuous variables were presented as mean or median, with data dispersion expressed as standard deviation (SD) or interquartile range (IQR), and were subsequently analyzed using the Student's t-test or the Mann-Whitney test to investigate differences between groups.

Comparisons between the ICU and non-ICU, IMV and non-IMV and COVID-19 severity (mild, moderate, and severe) groups were performed for each genotype and allele frequency. Contingency tables were created for genotypes (*e.g*., II *versus* ID *versus* DD) and alleles(*e.g*., I *versus* D). The *χ*^2^ test (or Fisher's exact test when the expected cell count was <5) was used to assess statistical significance. Odds ratios (ORs) and 95% confidence intervals (95%CIs) were calculated using multivariate logistic regression adjusted for age, cardiovascular disease, and diabetes.

Single-nucleotide polymorphism were considered predictor variables in logistic regression models for the outcomes ICU, IMV, acute kidney injury (AKI), death, cough, dyspnea, shallow breathing and fatigue. Regression models were evaluated using the Hosmer-Lemeshow test, and multivariate logistic regressions analyses were performed including the covariates: cardiovascular disease, diabetes, and age. P-values <0.05 were considered statistically significant.

We performed a post hoc power analysis to evaluate whether the sample size was adequate to detect differences in SNP genotype frequencies according to ICU admission among women. Based on the observed proportions of the GG genotype (60% in ICU *versus* 20% in non-ICU patients), and sample sizes of 30 and 25, respectively, we obtained a power of 87.7% (*α*=0.05, two-sided test), indicating a high probability of detecting the observed difference. All statistical analyses were performed using R (R Core Team, 2020).

## RESULTS

Cohort 1 (acute COVID-19) comprised 112 patients hospitalized at our center with SARS-CoV-2 infection. The most common comorbidities were cardiovascular disease (59.5%), diabetes (37.5%), obesity (35.1%), and oncohematological disease (18.2%). The main complication during hospitalization was acute kidney injury (AKI), which affected 31 (27.7%) patients. Twenty-nine (25.9%) patients died during hospitalization. Regarding disease severity, 53 patients (47.2%) were classified as having severe COVID-19. This group exhibited a higher mean age compared with the moderate/mild group, along with a higher prevalence of diabetes. The demographic and clinical characteristics of cohort 1 are described in table 1.

**Table 1:** Demographic and clinical characteristics of hospitalized patients in Cohort 1

		All (n=112)	ICU (n=63)	Non-ICU (n=49)	IMV (n=45)	Non-IMV (n=67)	Severe (n=53)	Moderate (n=18)	Mild (n=41)
Age, years (mean±SD)		57.4±17.5	59.6±17.6	54.8±17.29	62.2±14.8	54.1± 18.51	62.2±15.5^#^	54.2±16.1	53.5±19.4
Male, n (%)		47 (42.0)	30 (46.9)	19 (38.8)	22 (48.9)	27 (40.3)	25 (47.2)	8 (44.4)	15 (36.6)
White (self-reported), n (%)		42 (43.5)	24 (43.6)	15 (34.9)	18 (45.0)	25 (43.9)	23 (48.9)	7 (46.7)	14 (38.9)
Comorbidities, n (%)									
	Cardiovascular disease	66 (59.5)	42 (66.7)	25 (51.0)	30 (68.2)	36 (53.7)	20 (47.6)	3 (21.4)	9 (25.0)
	Diabetes	42 (37.5)	28 (43.8)	15 (30.6)	21 (46.7)	22 (32.8)	26 (49.1)^#^	7 (38.9)	9 (22.0)
	Obesity	39 (35.1)	25 (39.7)	14 (28.6)	20 (45.5)	19 (28.4)	22 (42.3)	5 (27.8)	11 (26.8)
	Oncohematological disease	20 (18.2)	14 (22.6)	7 (14.3)	10 (23.3)	11 (16.4)	11 (21.6)	6 (33.3)	4 (9.8)
	Immunosuppression	18 (16.2)	13 (20.6)	6 (12.2)	10 (22.7)	9 (13.4)	10 (19.2)	5 (27.8)	4 (9.8)
Complications during hospitalization, n (%)									
Acute kidney injury		31 (27.7)	28 (45.2)	3 (6.2)^#^	26 (60.5)	5 (7.5)^#^	25 (49.0)^#^	2 (11.1)	3 (7.5)
Death		29 (25.9)	27 (42.2)	2 (4.1)^#^	26 (57.8)	3 (4.5)^#^	26 (49.1)^#^	1 (5.6)	2 (4.9)

Data are presented as mean±SD or n (%). P values were calculated using the Mann-Whitney U or X^2^ tests for comparisons between ICU *versus* non-ICU, IMV *versus* non-IMV, and severe *versus* moderate/mild groups. Significant differences (p<0.05) are indicated by #.

Patients were stratified according to COVID-19 severity classification (mild, moderate and severe). ICU: intensive care unit; IMV: invasive mechanical ventilation; SD: standard deviation. Genotype frequencies for all SNPs in cohort 1 showed no significant deviation from Hardy-Weinberg equilibrium, except for *ACE2* in women for the ICU outcome. We first compared the distribution of all SNPs according to COVID-19 severity (severe, moderate, and mild). Significant associations were observed for *ACE* rs4646994, *ACE2* rs2285666, *IL1A* rs1800587, and *TNF* rs1800629 (Table 2).

The *ACE* rs4646994 ID genotype was more frequently observed in the severe COVID-19 group, as shown in table 2. In the model evaluating progression to severe COVID-19 (severe *versus* moderate/mild cases; adjusted for cardiovascular disease, diabetes, and age) the ID genotype was significantly associated with an increased risk of severe COVID-19 (OR=2.58; 95%CI=1.06-6.47; p=0.038), whereas the II genotype was associated with protection against severe disease (OR=0.37; 95%CI=0.14-0.90; p=0.032).

**Table 2:** Distribution of genotypes and alleles of selected SNPs according to COVID-19 severity classification (mild, moderate, and severe)

SNP		Severe (n=53)	Moderate (n=18)	Mild (n=41)	*p* value‡	OR (95% CI)*	p value^#^
*ACE* rs4646994							
	II	2 (3.8)	1 (5.6)	4 (9.8)	0.498	1.19 (0.14-8.31)	0.862
	ID	35 (66.0)	8 (44.4)	16 (39.0)	0.025^#^	2.58 (1.06-6.47)	0.038^#^
	DD	16 (30.2)	9 (50.0)	21 (51.2)	0.085	0.37 (0.14-0.90)	0.032^#^
	I	39 (36.8)	10 (27.8)	24 (29.3)	0.440	1.75 (0.91- 3.41)	0.095
	D	67 (63.2)	26 (72.2)	58 (70.7)	-	0.57 (0.29- 1.10)	0.095
*ACE2* rs2285666							
Men (n=47)							
	G	19 (76.0)	6 (75.0)	12 (80.0)	0.908	1.11 (0.35-3.61)	0.854
	A	6 (24.0)	2 (25.0)	3 (20.0)	0.908	0.90 (0.28-2.32)	0.854
Women (n=65)							
	GG	20 (71.4)	7 (70.0)	10 (38.5)	0.035^#^	4.20 (1.22-16.36)	0.028^#^
	GA	8 (28.6)	3 (30.0)	16 (61.5)	0.035^#^	0.24 (0.06-0.82)	0.028^#^
	AA	0	0	0	-	-	-
	G	48 (85.7)	17 (85.0)	36 (69.2)	0.635	2.86 (0.99-9.57)	0.065
	A	8 (14.3)	3 (15.0)	16 (30.8)	-	0.35 (0.10-1.01)	0.065
*IL1A* rs1800587							
	CC	19 (35.8)	9 (50.0)	18 (43.9)	0.515	0.79 (0.32-1.91)	0.599
	CT	34 (64.2)	6 (33.3)	14 (34.1)	0.006^#^	3.05 (1.26- 7.71)	0.015^#^
	TT	0 (0.00)	3 (16.7)	9 (22.0)	0.002^#^	-	-
	C	72 (67.9)	24 (66.7)	50 (61.0)	0.599	1.41 (0.75-2.71)	0.293
	T	34 (32.1)	12 (33.3)	32 (39.0)	-	0.71 (0.37-1.34)	0.293
*IL6* rs1800795							
	GG	31 (58.5)	12 (66.7)	29 (70.1)	0.458	0.64 (0.25-1.64)	0.329
	GC	21 (39.6)	4 (22.2)	10 (24.4)	0.191	2.37 (0.93-6.29)	0.075
	CC	1 (1.9)	2 (11.1)	2 (4.9)	0.258	-	-
	G	83 (78.3)	28 (77.8)	68 (82.9)	0.470	0.93 (0.43-2.03)	0.860
	C	23 (21.7)	8 (22.2)	14 (17.1)	-	1.07 (0.49-2.32)	0.860
*IL10* rs1800871							
	TT	24 (45.3)	8 (44.4)	16 (42.1)	0.955	1.16 (0.48-2.83)	0.739
	TC	25 (47.2)	9 (50.0)	15 (39.5)	0.687	0.90 (0.36-2.22)	0.814
	CC	4 (7.5)	1 (5.6)	7 (18.4)	0.190	0.88 (0.20-3.70)	0.866
	T	73 (68.9)	25 (69.4)	47 (57.3)	0.692	1.27 (0.67-2.42)	0.919
	C	33 (31.1)	11 (30.6)	29 (35.4)	-	0.97 (0.50-1.87)	0.919
*IL10* rs1800896							
	*AA*	5 (9.4)	3 (16.7)	5 (12.2)	0.702	0.36 (0.06-1.55)	0.192
	*GA*	27 (50.9)	7 (38.9)	22 (53.7)	0.569	1.20 (0.50- 2.87)	0.685
	*GG*	21 (39.6)	8 (44.4)	14 (34.1)	0.732	1.21 (0.49- 2.99)	0.673
	*A*	37 (34.9)	13 (36.1)	32 (40.2)	0.748	0.73 (0.38-1.38)	0.337
	*G*	69 (65.1)	23 (63.9)	50 (59.8)	-	1.37 (0.73-2.60)	0.337
*TNF* rs1800629							
	GG	42 (79.2)	17 (94.4)	36 (87.8)	0.240	0.16 (0.02-0.67)	0.025^#^
	GA	11 (20.8)	1 (5.6)	4 (9.8)	0.164	6.32 (1.49-43.64)	0.025^#^
	AA	0 (0.00)	0 (0.00)	1 (2.4)	0.417	-	-
	G	95 (89.6)	35 (97.2)	76 (92.7)	0.344	0.24 (0.05-0.86)	0.042^#^
	A	11 (10.4)	1(2.8)	6 (7.3)	-	4.15 (1.16-19.66)	0.042^#^
*UMOD* rs4293393							
	AA	36 (67.9)	10 (55.6)	24 (58.5)	0.519	0.95 (0.38-2.32)	0.909
	AG	14 (26.4)	7 (38.9)	15 (36.6)	0.462	1.00 (0.40-2.51)	0.996
	GG	3 (5.7)	1 (5.6)	2 (4.9)	0.985	1.32 (0.14-12.33)	0.794
	A	86 (81.1)	27 (75.0)	63 (76.8)	0.659	0.93 (0.45-1.96)	0.853
	G	20 (18.9)	9 (25.0)	19 (23.2)	-	1.07 (0.51-2.24)	0.853
*UMOD* rs13333226							
	AA	31 (58.5)	9 (50.0)	22 (53.7)	0.792	0.88 (0.36- 2.11)	0.780
	AG	17 (32.1)	7 (38.9)	17 (41.5)	0.629	0.91 (0.37-2.22)	0.829
	GG	5 5 (9.4)	2 (11.1)	2 (4.9)	0.630	2.45 (0.42-19.44)	0.336
	A	79 (74.5)	25 (69.4)	61 (74.4)	0.821	0.63 (0.32-1.25)	0.190
	G	27 (25.5)	11 (30.6)	21 (25.6)	-	1.58 (0.80-3.13)	0.190
*UMOD* rs12917707							
	GG	40 (75.5)	13 (72.2)	27 (65.9)	0.590	0.98 (0.38-2.55)	0.974
	GT	12 (22.6)	4 (22.2)	13 (31.7)	0.565	1.11 (0.42-2.97)	0.831
	TT	1 (1.9)	1 (5.6)	1 (2.4)	0.702	0.55 (0.02-6.83)	0.654
	G	92 (86.8)	30 (83.3)	67 (81.7)	0.624	0.76 (0.34-1.70)	0.505
	T	14 (13.2)	6 (16.7)	15 (18.3)	-	1.31 (0.59-2.92)	0.505

Data are expressed as n (%).

‡ Comparisons were made between severity groups (mild, moderate, and severe) for each genotype and allele frequency. Contingency tables were constructed for genotypes (*e.g.*, II versus ID *versus* DD) and alleles (*e.g.*, I *versus* D), and the χ2 test (or Fisher's exact test when expected cell counts were <5) was used to assess statistical significance;

* Odds ratios (ORs) and 95% confidence intervals (95%CIs) were calculated using multivariate logistic regression adjusted for age, cardiovascular disease and diabetes, with severe COVID-19 as the outcome. Data were stratified by sex for ACE2 rs2285666; # Statistically significant values (p<0.05).

In females, the GG genotype of *ACE2* rs2285666 was more frequent in severe cases (Table 2). Multivariate logistic regression confirmed that this genotype was associated with an increased risk risk of severe COVID-19 in females compared with non-severe cases (OR=4.20; 95%CI=1.22-16.36; p=0.028).

The *IL1A* rs1800587 AA genotype was more frequently observed among patients with mild disease, as described in table 2. In the model evaluating progression to severe COVID-19, the *IL1A* CT genotype was significantly associated with an increased risk of developing severe disease (OR=3.05; 95%CI=1.26- 7.71; p=0.015).

The *TNF* rs1800629 GA genotype was also more frequent in severe cases of COVID-19 (Table 2). When compared with non-severe cases in the multivariate logistic regression analysis, the GA genotype (OR=6.32, 95%CI=1.49-43.64; p=0.025) and the A allele (OR=4.15; 95%CI=1.16-19.66; p=0.042) were associated with an increased risk of developing severe disease.

**Figure 1:**
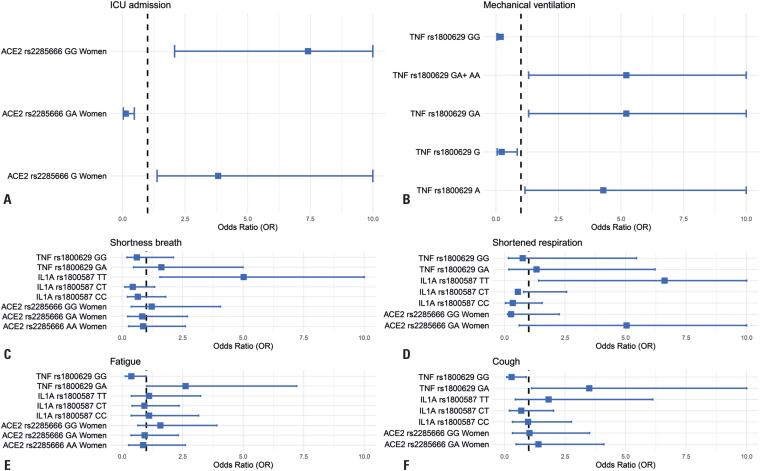
The graphs in the first row represent (A) ICU admission and (B) the need for invasive mechanical ventilation in patients with acute COVID-19. The second row includes outcomes related to the post COVID-19 phase, including (C) dyspnea, (D) shallow breathing, (E) fatigue, and (F) cough. Each blue dot represents the estimated OR, with horizontal bars indicating the 95% confidence interval (95%CI), which was truncated at 10 for better visualization

According to ICU admission, we identified that the *ACE2* rs2285666 showed statistically significant differences between groups, but only among women. In the multivariate logistic regression analysis, we observed that the *ACE2* GG genotype was more frequent in women admitted to the ICU (p=0.021) (Table 3) with a significant risk effect (OR=7.41, 95%CI=2.08-31.68, p=0.003), whereas the GA genotype was more frequent in non-ICU patients (p=0.021) (OR=0.13, 95%CI=0.03-0.47, p=0.003) (Figure 1A). The G allele also showed a significant risk effect (OR=3.82, 95%CI=1.38-11.89, p=0.013) in the multivariate logistic regression analysis, whereas the A allele showed a protective effect (OR=0.26, 95%CI=0.08-0.72, p=0.013) for ICU admission in women (Figure 1A). No associations were observed for the other genes.

**Table 3:** Distribution of genotypes and alleles of selected single-nucleotide polymorphisms according to intensive care unit admission

SNP		Non-ICU (n=49)	ICU (n=63)	p value#	OR (95% CI)	p value#
*ACE rs4646994*						
	II	2 (4.1)	6 (9.5)	0.460	2.65 (0.51-19.9)	0.052
	ID	26 (53.1)	32 (50.8)	0.962	0.79 (0.34-1.85)	0.599
	DD	21 (42.8)	25 (39.7)	0.885	0.97 (0.41-2.29)	0.944
	I	30 (30.6)	44 (34.9)	0.591	1.17 (0.62-2.20)	0.622
	D	68 (69.4)	82 (65.1)	-	0.85 (0.45-1.59)	-
*ACE2* rs2285666						
	Men (n=47)					
	G	14 (73.7)	22 (78.6)	0.970	1.84 (0.33-11.57)	0.491
	A	5 (26.3)	6 (21.4)	-	0.54 (0.08-3.03)	-
Women (n=65)						
	GG	12 (40.0)	25 (71.4)	0.021*	7.41 (2.08-31.68)	0.003*
	GA	18 (60.0)	10 (28.6)	0.021*	0.13 (0.03-0.47)	0.003*
	AA	0	0	-	-	-
	G	42 (70.0)	60 (85.7)	0.069	3.82 (1.38-11.89)	0.013*
	A	18 (30.0)	10 (14.3)	-	0.26 (0.08-0.72)	-
*IL1A* rs1800587						
	CC	18 (36.7)	27(42.8)	0.812	0.58 (0.18-1.79)	0.354
	CT	22 (44.8)	33 (52.4)	0.552	1.13 (0.48-2.63)	0.777
	TT	7 (14.2)	5 (7.9)	0.441	0.58 (0.18-1.79)	0.354
	C	62 (63.3)	83 (65.9)	0.888	1.23 (0.66-2.28)	0.500
	T	36 (36.7)	43 (34.1)	-	0.81 (0.44-1.50)	-
*IL6* rs1800795						
	GG	34 (69.4)	37 (58.7)	1.000	2.62 (0.24-1.53)	0.305
	GC	13 (26.5)	23 (36.5)	0.359	2.01 (0.78-5.4)	0.152
	CC	2 (4.1)	3 (4.8)	0.335	0.27 (0.011-3.41)	0.328
	G	81 (82.7)	97 (77.0)	0.381	0.69 (0.32-1.48)	0.351
	C	17 (17.3)	29 (23.0)	-	1.43 (0.67-3.11)	-
*IL10* rs1800871						
	TT	20 (43.5)	27 (42.9)	1.000	1.47 (0.62-3.58)	0.381
	TC	20 (43.5)	30 (47.6)	0.815	0.98 (0.4-2.36)	0.968
	CC	6 (13.0)	6 (9.5)	0.787	0.52 (0.12-2.08)	0.359
	T	60 (65.2)	84 (66.7)	0.938	1.37 (0.73-2.61)	0.320
	C	32 (34.8)	42 (33.3)	-	0.72 (0.38-1.36)	-
*IL10* rs1800896						
	*AA*	6 (12.2)	7 (11.1)	1	0.92 (0.22-3.72)	0.901
	*GA*	25 (51.0)	30 (47.6)	0.868	0.96 (0.41-2.25)	0.928
	*GG*	18 (36.7)	26 (41.3)	0.77	1.08 (0.45-2.61)	0.862
	*A*	37 (37.8)	44 (34.9)	0.65	0.87 (0.47-1.63)	0.670
	*G*	61 (62.2)	82 (65.1)	-	1.14 (0.61-2.13)	-
*TNF* rs1800629						
	GG	45 (91.8)	50 (79.4)	1	0.38 (0.07-1.45)	0.181
	GA	4 (8.2)	12 (19.0)	0.174	2.62 (0.69-12.79)	0.181
	AA	0 (0.0)	1 (1.6)	0.119	-	-
	G	94 (95.9)	112 (88.9)	0.094	0.40 (0.08-1.44)	0.538
	A	4 (4.1)	14 (11.1)	-	2.49 (0.69-11.74)	0.191
*UMOD* rs4293393						
	AA	29 (59.2)	40 (63.5)	0.788	2.10 (0.82-5.47)	0.122
	AG	15 (30.6)	22 (34.9)	0.781	1.7 (0.68-4.28)	0.257
	GG	5 (10.2)	1 (1.6)	0.113	0.27 (0.03-1.42)	0.141
	A	73 (74.5)	1102 (81.0)	0.318	1.16 (0.57-2.37)	0.670
	G	25 (25.5)	24 (19.0)	-	0.85 (0.42-1.75)	-
*UMOD* rs13333226						
	AA	26 (53.1)	35 (55.5)	0.943	0.86 (0.36-2.02)	0.739
	AG	17 (34.7)	25 (39.7)	0.731	1.55 (0.64-3.83)	0.328
	GG	6 (12.2)	3 (4.8)	0.274	0.31 (0.039-1.84)	0.219
	A	69 (70.4)	95 (75.4)	0.494	1.04 (0.53-2.03)	0.908
	G	29 (29.6)	31 (24.6)	-	0.96 (0.49-1.88)	-
*UMOD* rs12917707						
	GG	32 (65.3)	47 (74.6)	0.389	1.59 (0.70-3.63)	0.263
	GT	14 (28.6)	16 (25.4)	0.872	0.76 (0.32-1.79)	0.534
	TT	3 (6.1)	0	0.161	-	-
	G	78 (79.6)	110 (87.3)	0.169	1.61 (0.73-3.61)	0.240
	T	20 (20.4)	16 (12.7)	-	0.62 (0.27-1.36)	-

Data are expressed as n (%).

# Comparisons were made between intensive care unit and non-intensive care unit patients for each genotype and allele frequency. Contingency tables were constructed for genotypes (*e.g.*, II *versus* ID *versus* DD) and alleles (*e.g*., I *versus* D), and the *χ*^2^ test (or Fisher's exact test when expected cell counts were <5) was used to assess statistical significance. Odds ratios (ORs) and 95% confidence intervals (95%CIs) were calculated using multivariate logistic regression adjusted for age, cardiovascular disease, and diabetes. Data were stratified by sex for ACE2 rs2285666;

* Statistically significant values (p<0.05). ICU: intensive care unit.

Next, we compared the frequency of SNPs in COVID-19 patients who required IMV and those who did not (non-IMV). Regarding IMV, we identified that *IL1A* rs1800587 and *TNF* rs1800629 showed statistically significant differences (Table 4). The *IL1A* CT genotype was more frequent in patients receiving IMV (p=0.016), whereas the TT genotype was more frequent in non-IMV patients (p=0.007). However, we found no difference in the multivariate logistic regression analysis for any *IL1A* genotypes or alleles. The polymorphic A allele of rs1800629 in *TNF* showed a risk effect (OR=4.29; 95%CI=1.16-20.54; p<0.001), whereas the G allele showed a protective effect (OR=0.23; 95%CI=0.05-0.85; p=0.039) (Figure 1B). In a dominant model of the A allele, the GA+AA genotypes showed a risk effect for IMV (OR=5.21; 95%CI=1.31-26.44; p=0.026).

**Table 4:** Distribution of genotypes and alleles of selected single-nucleotide polymorphisms according to invasive mechanical ventilation requirement

SNP		Non-IMV (n=66)	IMV (n=45)	p value#	OR (95% CI)	p value#
*ACE* rs4646994						
	II	2 (3.0)	6 (13.3)	0.092	6.24 (1.07-51.1)	0.522
	ID	35 (53.0)	23 (51.1)	0.996	1.04 (0.41-2.61)	0.924
	DD	29 (44.0)	16 (35.6)	0.492	0.56 (0.21-1.43)	0.234
	I	39 (29.5)	35 (38.9)	0.192	1.76 (0.90-3.47)	0.096
	D	93 (70.5)	55 (61.1)	-	0.56 (0.28-1.10)	-
*ACE2* rs2285666						
	Men (n=47)					
	G	20 (80.0)	16 (72.7)	0.918	0.86 (0.14-5.42)	0.872
	A	5 (20.0)	6 (27.3)	0.918	1.15 (0.18-6.94)	0.872
Women (n=65)						
	GG	21 (52.5)	15 (65.2)	0.473	0.85 (0.33-2.20)	0.746
	GA	19 (47.5)	8 (34.8)	0.473	1.59 (0.61-4.31)	0.344
	AA	0	0	-	-	-
	G	61 (76.2)	38 (82.6)	0.77	1.86 (0.62-6.35)	0.283
	A	19 (23.8)	8 (17.4)	-	0.53 (0.15-1.58)	-
*IL1A* rs1800587						
	CC	28 (42.4)	16 (35.6)	0.597	0.42 (0.1-1.45)	0.190
	CT	26 (39.4)	29 (64.4)	0.016*	1.57 (0.63-3.98)	0.336
	TT	12 (18.2)	0 (0.0)	0.007*	1.38 (0.48-4.01)	0.539
	C	82 (62.1)	61 (67.8)	0.582	1.46 (0.75-2.91)	0.268
	T	50 (37.9)	29 (32.2)	-	0.68 (0.34-1.33)	-
*IL6* rs1800795						
	GG	44 (66.7)	26 (57.8)	0.452	0.66 (0.25-1.77)	0.413
	GC	18 (27.3)	18 (40.0)	0.23	2.31 (0.83-6.63)	0.111
	CC	4 (6.1)	1 (2.2)	0.623	0.58 (NA-6.37)	0.989
	G	106 (80.3)	70 (77.8)	0.774	0.86 (0.38-1.93)	0.712
	C	26 (19.7)	20 (22.2)	-	1.16 (0.52-2.57)	-
*IL10* rs1800871						
	TT	27 (42.9)	20 (44.4)	1	1.1 (0.43-2.81)	0.837
	TC	27 (42.9)	22 (48.9)	0.671	1.08 (0.41-2.8)	0.873
	CC	9 (14.3)	3 (6.7)	0.352	0.74 (0.13-3.29)	0.709
	T	81 (64.3)	62 (68.9)	0.576	1.22 (0.62-2.43)	0.568
	C	45 (35.7)	28 (31.1)	-	0.82 (0.41-1.61)	-
*IL10* rs1800896						
	AA	9 (13.6)	4 (8.9)	0.643	0.25 (0.03-1.28)	0.128
	AG	31 (47.0)	23 (51.1)	0.814	1.38 (0.55-3.54)	0.489
	GG	26 (39.4)	18 (40.0)	1	1.15 (0.44-2.96)	0.77
	A	49 (37.1)	31 (34.5)	-	0.75 (0.38-1.48)	-
	G	83 (62.9)	59 (65.5)	0.924	1.31 (0.67-2.62)	0.424
*TNF* rs1800629						
	GG	59 (89.4)	35 (77.8)	0.161	0.19 (0.04-0.76)	0.026*
	GA	6 (9.1)	10 (22.2)	0.097	5.21 (1.31-26.44)	0.026*
	AA	1 (1.5)	0 (0.0)	1		
	GA+AA	7 (10.6)	10 (22.2)	0.098	5.21 (1.31-26.44)	0.026*
	G	124 (93.9)	80 (88.9)	0.270	0.23 (0.05-0.85)	0.039*
	A	8 (6.1)	10 (11.1)	-	4.29 (1.16-20.54)	<0.001*
*UMOD* rs4293393						
	AA	38 (57.6)	30 (66.7)	0.443	0.85 (0.33-2.20)	0.746
	AG	23 (34.8)	14 (31.1)	0.838	1.59 (0.61-4.31)	0.344
	GG	5 (7.6)	1 (2.2)	0.425	0.51 (0.06-2.89)	0.474
	A	99 (75.0)	74 (82.2)	0.267	1.13 (0.52-2.49)	0.754
	G	33 (25.0)	16 (17.8)	-	0.88 (0.40-1.90)	-
*UMOD* rs13333226						
	AA	35 (53.0)	25 (55.6)	0.946	0.8 (0.31-2.03)	0.639
	AG	25 (37.9)	17 (37.8)	1	1.45 (0.56-3.85)	0.446
	GG	6 (9.1)	3 (6.7)	0.916	0.58 (0.065-3.75)	0.587
	A	95 (72.0)	67 (74.4)	0.8	0.91 (0.44-1.89)	0.801
	G	37 (28.0)	23 (25.6)	-	1.09 (0.52-2.24)	-
*UMOD* rs12917707						
	GG	44 (66.7)	34 (75.6)	0.427	1.04 (0.38-2.93)	0.924
	GT	19 (28.8)	11 (24.4)	0.773	1.07 (0.37-3.03)	0.129
	TT	3 (4.5)	0 (0.0)	0.393	-	-
	G	107 (81.1)	79 (87.8)	0.251	1.24 (0.53-3.06)	0.617
	T	25 (18.9)	11 (12.2)	-	0.80 (0.32-1.87)	-

Data are expressed as n (%).

# Comparisons were made between IMV and non-IMV patients for each genotype and allele frequency. Contingency tables were constructed for genotypes (e.g., II *versus* ID *versus* DD) and alleles (*e.g.*, I *versus* D), and the χ^2^ test (or Fisher's exact test when expected cell counts were <5) was used to assess statistical significance. Odds ratios (ORs) and 95% confidence intervals (95%CIs) were calculated using multivariate logistic regression adjusted for age, cardiovascular disease, and diabetes. Data were stratified by sex for ACE2 rs2285666

* Statistically significant values (p<0.05). IMV: invasive mechanical ventilation.

Based on the impact of *TNF, ACE2,* and *IL1A* polymorphisms on specific outcomes in patients with acute COVID-19 (ICU admission and need for IMV), we analyzed these SNPs according to symptoms and PCC development. In this analysis, cohort 2 (n=107 patients) had a mean age of 54.70±15.18 years, and 73.8% were female. The most frequent comorbidities were hypertension (56.1%), dyslipidemia (47.7%), diabetes (30.8%), and depression (28.0%). The most commonly reported symptoms in the post-COVID-19 period were persistent cough (15.9%), dyspnea (16. 8%), shallow breathing (7.5%), and exertional fatigue (44.9%). The clinical and epidemiological data of the patients in the post-COVID-19 period are shown in the Table 1S, Supplementary Material.

The genotype frequencies of all the SNPs in cohort 2 did not deviate from the Hardy-Weinberg equilibrium. We then analyzed the SNPs associated with cough using regression analysis (Figure 1F), in which the *TNF* GA genotype showed a significant risk effect for the persistence of this symptom (OR=3.50, 95%CI=1.12-10.67, p=0.03), whereas the GG genotype showed a protective effect (OR=0.29, 95%CI=0.09-0.86, p=0.03). For the symptom of exertional fatigue (Figure 1E), we obtained similar results, the GG genotype showed protective effect (OR=0.38, 95%CI=0.13-0.98, p=0.049) whereas the GA genotype showed a risk effect (OR=2.62, 95%CI=1.01-7.91; p=0.049). For the other symptoms, no relevant associations were observed, corroborating the findings from the acute COVID-19 analysis and suggesting that the *TNF r*s1800629 polymorphism is associated with the course of the disease. In addition, the TT genotype of *IL1A* was identified as a risk factor for symptoms of dyspnea (Figure 1C) (OR =5.02; 95%CI 1.56-16.07; p =0.006) and shallow breathing (Figure 1D) (OR =6.61; 95%CI 1.41-31.30; p =0.013), suggesting a possible relationship between this SNP and the persistence of symptoms however no association was observed between with exertional fatigue and cough. *ACE2* (rs2285666) was not statistically significant regarding the persistence of reported symptoms.

Regarding the haplotype analysis, complete linkage disequilibrium (D’ =1) was detected between *IL10* rs1800871 and rs1800896. The allele combinations observed in our total sample were T-A (36.2%), C-G (34.0%), and T-G (29.8%). For *UMOD* polymorphisms, rs12917707 was in complete linkage disequilibrium with rs13333226 and rs4293393. *UMOD* rs13333226 and rs4293393 were almost in complete linkage disequilibrium (D’ =0.971). The *UMOD haplotypes* (rs12917707-rs13333226-rs4293393) showed a higher frequency of the G-A-A haplotype (73.0%), followed by T-G-G (15.8%), while the remaining haplotypes (G-A-G, G-G-A, G-G-G) accounted for 11.2% of the haplotype distribution in the total sample (Table 2S, Supplementary Material). We compared the haplotypes with clinical outcomes, including hospitalization, ICU admission, and IMV, however, no associations were observed. The complete results are provided in the Tables 3S, 4S and 5S, Supplementary Material.

## DISCUSSION

The mechanisms involved in the pathogenesis of COVID-19 have already been widely explored. However, some aspects of the acute and post-acute phases of the disease remain poorly understood. As previously reported, variations in a single nucleotide in the genome sequence may explain individual clinical susceptibility to COVID-19.^(14)^ In this study we aimed to investigate whether SNPs in different genes, *including IL1A* (rs1800587), *IL6* (rs1800795), *IL10* (rs1800896 and rs1800871), *TNF* (rs1800629), *ACE* (rs4646994), *ACE2* (rs2285666) and *UMOD* (rs4293393, rs13333226 and rs12917707) were associated with the COVID-19 severity during the acute phase or with the persistence or emergence of symptoms in the post-COVID phase in Brazilian patients.

ACE and its homolog, ACE2, are two essential enzymes responsible for generating bioactive peptides within the renin-angiotensin system (RAS).^(33)^ Both ACE and ACE2 play pivotal roles in regulating inflammatory processes.^(34)^ The I allele of *ACE* rs4646994 (I/D) has been associated with reduced circulating levels of ACE, potentially due to the inhibition of RNA polymerase II-mediated mRNA transcription or alternative splicing mechanisms that produce a truncated ACE protein lacking one of its active sites.^(35,36)^ In your study, we found a significant association between the DD genotype and protection against severe COVID-19. Yenmis et al.^(37)^ also reported that the DD genotype and protective against severe SARS-CoV-2. However, previous studies have shown conflicting results,^(33,38)^ while others did not find this association.^(39,40)^ Alimoradi el al.^(22)^ observed an association between *ACE* and susceptibility to COVID-19 infection, but not with disease severity. Therefore, further studies are needed to clarify the true impact of this polymorphism on COVID-19 severity.

*ACE2* encodes the primary receptor for SARS-CoV-2 entry into host cells. The rs2285666 polymorphism in *ACE2* is a splice region variant and has been shown to increase serum levels of ACE2,^(41)^ potentially facilitating viral entry and increasing disease susceptibility and severity.^(42)^ In our study, the G allele of *ACE2* (particularly the GG genotype) was associated with a higher risk of COVID-19 and ICU admission in women. The *ACE2* rs2285666 polymorphism has previously been associated with hypertension.^(22,43)^ Therefore, it is important to note that we did not observe a difference in the frequency of hypertension between sexes (p=0.351). Möhlendick et al. reported that patients carrying the G allele or the GG genotype had a threefold increased risk of developing severe COVID-19.^(41)^ On the other hand, results from a recent meta-analysis did not confirm this association.^(14, 38)^

Nevertheless, the variability in these results may be attributed to differences in clinical characteristics, epigenetic mechanisms regulating *ACE2* receptor expression, or variations in other genes, such as those involved in immune response or coagulation pathways, which may influence disease prognosis.^(44)^ Furthermore, it is important to note that we did not observe an association between this SNP and the persistence or development of long-term symptoms. Similarly, Fernández-de-Las-Peñas et al. found no significant association with symptom persistence in individuals who had previously been hospitalized, but only with the severity of the acute infection.^(45)^

In the present study, we also identified two genes associated with with COVID-19 severity and the need for IMV as clinical outcomes, *IL1A* (rs1800587, CT genotype) and *TNF* (rs1800629, GA+AA genotypes). The rs1800587 polymorphism is located in the promoter region of the *IL1A* and has been associated with increased IL-1*α* expression, which may intensify the inflammatory response, and create a favorable environment for chronic inflammation.^(46)^ When we performed the multivariate analysis controlling for potential confounders, we confirmed the significant association between *IL1A* rs1800587 and COVID-19 severity, but not with the need for IMV. To our knowledge, there are no studies investigating SNP in *IL1A* and the need for IMV in patients with COVID-19.^(47)^ However, in our analysis of the post-COVID period, *IL1A* rs1800587 was significantly associated with dyspnea and shortened of breath. The literature has already described a relationship between *IL1A* rs1800587 and diseases caused by other coronaviruses.^(48)^These findings highlight the importance of further investigating how genetic variations influence recovery and potential complications after COVID-19, as studies addressing the association of this SNP with the development of PCC remain scarce.

Lastly, rs1800629, located in the promoter region of the *TNF* gene, is associated with increased production of TNF-*α* and may exacerbate systemic inflammation.^(49,50)^ In the present study, the A-allele of this SNP was associated with a higher risk of severe COVID-19 and the need for IMV, while the G allele showed a protective effect. When analyzing genotypes, this association was further supported, as patients carrying the GG genotype showed a reduced risk of severe COVID-19 or requiring IMV. The *TNF* gene is located within the class III region of the major histocompatibility complex (MHC) on chromosome 6p21.3.^(51)^ TNF-*α* is well-known for its pro-inflammatory properties and, together with IL-1 and IL-6, plays an important role in the inflammatory response to COVID-19.^(52-54)^ Moreover, the rs1800629 has been associated with sepsis.^(55,56)^ A recent meta-analysis evaluating different *TNF* polymorphisms found a higher frequency of the AA and GA genotypes among patients who required IMV in COVID-19, however this difference was not statistically significant.^(56)^ In a study conducted in Egypt in 2020, individuals carrying the A-allele (GA and AA genotypes) were more susceptible to COVID-19, and the AA genotype was associated with severe disease and the need for IMV.^(9)^ Importantly, it has been documented that the A allele of in the *TNF* promoter region (rs1800629) leads to increased transcriptional activity, resulting in elevated production of TNF-*α* by B and T cells.^(57)^ Consistent with our findings, these results suggest that the association of the A allele with severe COVID-19 may be explained by a dysregulated TNF-*α* production.

We also observed an association between *TNF* rs1800629 and PCC. In the study of Fernández-de-Las-Peñas et al, no association between rs1800629 and post-COVID-19 symptoms was observed.^(58)^ However, our findings suggest that this SNP influences both the acute phase and symptom persistence, particularly in the respiratory tract. This is supported by evidence linking TNF-*α* to asthma, acute respiratory distress syndrome, and chronic obstructive pulmonary disease, conditions in which TNF-*α* is directly involved in inflammatory processes affecting the alveoli and bronchi.^(59)^ Furthermore, as reported by Saleh et al, the A allele and the GA genotype of *TNF* are directly associated with acute COVID-19, however, our findings suggest that they are also associated with symptom persistence in the post-COVID phase, as this allele leads to increased transcriptional activity and elevated production of TNF-*α* by B and T cells.^(49,57)^

In this study, we did not aim to evaluate the combined effect of SNPs and comorbidities on COVID-19 severity, however, previous studies have demonstrated associations between genetic variations and comorbidities strongly related to COVID-19. Data from genome-wide association studies (GWAS) have identified genetic variants associated with COVID-19 severity in patients with asthma, obesity, and type 2 *diabetes mellitus*.^(60,61)^ Other case-control studies have reported associations between SNPs, and increased risk of hypertension, obesity, diabetes, and dyslipidemia in the context of COVID-19.^(62,63)^ Shared molecular mechanisms underlying the co-occurrence of COVID-19 and conditions such as osteoarthritis, obesity, and type 2 diabetes have also been investigated.^(64-67)^ Therefore, a combination of multiple genes and non-genetic factors is likely involved in COVID-19 severity and its associated comorbidities. Elucidating reliable markers of COVID-19 severity in patients with comorbidities is crucial for developing personalized treatments strategies.

This study has some limitations, mainly the relatively sample size. In addition, our cohort primarily consisted of patients with multiple comorbidities, as our center is a tertiary referral hospital. Furthermore, comparison with a healthy control group was not possible. Likewise, despite these limitations, our study identified associations between genes related to cytokines and renin-angiotensin-aldosterone system (RAAS) and COVID-19 severity, as well as the persistence of symptoms in the post-COVID-19 period.

## CONCLUSION

Our findings indicate an important role for variations in *ACE2, IL1A,* and *TNF* in the risk of developing severe COVID-19, as well for *IL1A* and *TNF* in the persistence of symptoms in the post-COVID-19 phase among Brazilian patients. The A allele of *TNF* rs1800629 may represent a risk variant for disease severity, the need for IMV during the acute phase of COVID-19, and the persistence of cough and fatigue, suggesting a link between acute disease severity post-COVID-19 condition. The *IL1A* rs1800587 polymorphism was associated with disease severity during the acute phase and with the presence of dyspnea and shallow breathing after COVID-19. In contrast, ACE2 polymorphisms in women were associated with disease severity, but not with symptom persistence.

## Data Availability

The underlying content is contained within the manuscript.
