# Supplementary material for: Influence of inflammatory and reninangiotensin system gene polymorphisms ACE2 rs2285666, IL1A rs1800587, and TNF rs1800629 on COVID-19 severity and the persistence of symptoms in the post-COVID-19 phase: a cross-sectional study
Source: Einstein (Sao Paulo). 2026 May 21;24:eAO1503. doi: 10.31744/einstein_journal/2026AO1503 (PMC13399304; doi:10.31744/einstein_journal/2026AO1503)
Supplement: Supplementary Material [file 2317-6385-eins-24-eAO1503-Suppl01.pdf]

SUPPLEMENTARY MATERIAL

Influence of inflammatory and renin-angiotensin system gene polymorphisms *ACE2* rs2285666, *IL1A* rs1800587, and *TNF* rs1800629 on COVID-19 severity and the persistence of symptoms in the post-COVID-19 phase: a cross-sectional study

Matheus Daudt-Lemos, Evelyn Maciel de Oliveira, Alice Ramos-Silva, Natalia Fonseca Rosário, Thays Araújo Gonçalves, Camila de Melo Carvalho Nascimento, Amanda Mendes do Valle, Lialyz Soares Pereira André, Fabio Aguiar-Alves, Jorge Paulo Strogoff de Matos, Jocemir Ronaldo Lugon, Jorge Reis Almeida, Thalia Medeiros, Fabiana Barzotto Kohlrausch, Andrea Alice Silva

DOI: 10.31744/einstein_journal/2026AO1503

Table 1S. Demographic and clinical characteristics of patients in the post-COVID-19 period

Characteristics	All (n=107)
Age (years, mean±SD)	54.70±15.18
Female, n (%)	79 (73.8)
White (self-declared), n (%)	42 (39.3)
Comorbidities, n (%)	
Diabetes	33 (30.8)
Depression	30 (28.0)
Hypertension	60 (56.1)
Dyslipidemia	51 (47.7)
Post-COVID symptoms, n (%)	
Cough	17 (15.9)
Dyspnea	18 (16.8)
Shallow breathing	8 (7.5)
Exertional fatigue	48 (44.9)

Data are presented as mean±SD (standard deviation) or number (%).

Table 2S. Haplotype frequencies of *IL10* and *UMOD* in the study population

Haplotypes	n (%)
IL10 (rs1800871-rs1800896)	
C-G	73 (34.0)
T-A	79 (36.2)
T-G	64 (29.8)
UMOD (rs12917707-rs13333226-rs4293393)	
G-A-A	164 (73.2)
G-A-G	1 (0.4)
G-G-A	12 (5.4)
G-G-G	12 (5.4)
T-G-G	35 (15.6)

Data are presented as numbers (%).

Table 3S. Distribution of *IL10* and *UMOD* haplotypes according to intensive care unit admission

Haplotypes	Non-ICU n (%)	ICU n (%)	p value
IL10 (rs1800871-rs1800896)			0.719
C-G	32 (35.6)	41 (32.5)	
T-A	34 (37.8)	45 (35.7)	
T-G	24 (26.7)	40 (31.7)	
UMOD (rs12917707-rs13333226-rs4293393)			0.316
G-A-A	68 (69.4)	96 (76.2)	
G-A-G	1 (1.0)	0 (0.0)	
G-G-A	5 (5.1)	7 (5.6)	
G-G-G	5 (5.1)	8 (6.3)	
T-G-G	20 (20.4)	15 (11.9)	

Data are presented as n (%). Comparison of haplotype frequencies between patients admitted and not admitted to the ICU. were performed using the X^2 or Fisher's exact test. P-value <0.05 was considered statistically significant.
ICU: intensive care unit.

Table 4S. IL10 and UMOD haplotype frequencies according to the requirement for invasive mechanical ventilation

Haplotypes	Non-IVM n (%)	IVM n (%)	p value
IL10 (rs1800871-rs1800896)			0.515
C-G	45 (35.7)	27 (30.7)	
T-A	47 (37.3)	31 (35.2)	
T-G	34 (27.0)	30 (34.1)	
UMOD (rs12917707-rs13333226-rs4293393)			0.366
G-A-A	96 (71.6)	66 (75.0)	
G-A-G	1 (0.7)	0 (0.0)	
G-G-A	5 (3.7)	7 (8.0)	
G-G-G	7 (5.2)	5 (5.7)	
T-G-G	25 (18.7)	10 (11.4)	

Data are presented as n (%). Comparison of haplotype frequencies according to the requirement for invasive mechanical ventilation using the X^2 or Fisher's exact tests. A p<0.05 was considered statistically significant.
IVM: invasive mechanical ventilation.

Table 5S. Frequencies of *IL10* and *UMOD* haplotypes stratified by COVID-19 severity

Haplotypes	Mild n (%)	Moderate n (%)	Severe n (%)	p value
IL10 (rs1800871-rs1800896)				0.085
C-G	30 (40.5)	11 (30.6)	32 (30.2)	
T-A	31 (41.9)	11 (30.6)	37 (34.9)	
T-G	13 (17.6)	14 (38.9)	37 (34.9)	
UMOD (rs12917707-rs13333226-rs4293393)				0.817
G-A-A	6 (16.7)	25 (69.4)	79 (74.5)	
G-A-G	1 (1.2)	0 (0.0)	0 (0.0)	
G-G-A	3 (3.7)	2 (5.6)	7 (6.6)	
G-G-G	3 (3.7)	3 (8.3)	6 (5.7)	
T-G-G	15 (18.3)	6 (16.7)	14 (13.2)	

Data are presented as numbers (%). Haplotype frequencies were compared among patients according to COVID-19 severity (mild, moderate, or severe/critical) using the X^2 test or Fisher's exact tests. P-value <0.05 was considered statistically significant.
